# Nanotube Action between Human Mesothelial Cells Reveals Novel Aspects of Inflammatory Responses

**DOI:** 10.1371/journal.pone.0029537

**Published:** 2011-12-27

**Authors:** Julia Ranzinger, Amin Rustom, Marcus Abel, Julia Leyh, Lars Kihm, Margarete Witkowski, Peter Scheurich, Martin Zeier, Vedat Schwenger

**Affiliations:** 1 Department of Nephrology, University of Heidelberg, Heidelberg, Germany; 2 Department of New Materials and Biosystems, Max Planck Institute for Intelligent Systems, Stuttgart, Germany; 3 Department of Biophysical Chemistry, University of Heidelberg, Heidelberg, Germany; 4 Institute of Cell Biology and Immunology, University of Stuttgart, Stuttgart, Germany; Charité-University Medicine Berlin, Germany

## Abstract

A well-known role of human peritoneal mesothelial cells (HPMCs), the resident cells of the peritoneal cavity, is the generation of an immune response during peritonitis by activation of T-cells *via* antigen presentation. Recent findings have shown that intercellular nanotubes (NTs) mediate functional connectivity between various cell types including immune cells - such as T-cells, natural killer (NK) cells or macrophages - by facilitating a spectrum of long range cell-cell interactions. Although of medical interest, the relevance of NT-related findings for human medical conditions and treatment, *e.g.* in relation to inflammatory processes, remains elusive, particularly due to a lack of appropriate *in vivo* data. Here, we show for the first time that primary cultures of patient derived HPMCs are functionally connected *via* membranous nanotubes. NT formation appears to be actin cytoskeleton dependent, mediated by the action of filopodia. Importantly, significant variances in NT numbers between different donors as a consequence of pathophysiological alterations were observable. Furthermore, we show that TNF-α induces nanotube formation and demonstrate a strong correlation of NT connectivity in accordance with the cellular cholesterol level and distribution, pointing to a complex involvement of NTs in inflammatory processes with potential impact for clinical treatment.

## Introduction

Chronic inflammatory processes lead to the impairment of tissue integrity. This problem is exemplified by peritoneal dialysis (PD), which over the past few years attained increased relevance as continuous renal replacement therapy. The implantation and presence of an indwelling catheter, the dialysis solution itself, as well as peritonitis - a known complication of PD - coincide with high levels of proinflammatory cytokines within the peritoneal cavity [1]–[3].

In context of inflammatory immune reactions, intercellular communication plays a fundamental role. The recent awareness that eukaryotic cells can be linked *via* membrane tubes, facilitating the intercellular transmission of electric signals [4] or various cellular components [5], [6], has extended previous conceptions of cell-to-cell communication. Apart from mediating functional connectivity between cells of the immune system, *e.g.* T-cells [7], natural killer cells [8] or macrophages [9], there is culminating evidence for a participation of NTs in several pathological processes of substantial medical interest. Although few publications have proven the existence of NTs *in vivo* [10], their occurrence, architecture and function in the body is still a matter of considerable debate. Facing this background, we were interested whether NTs are formed in human peritoneum and - if so - whether their occurrence correlates with defined pathophysiological conditions.

## Results

### Nanotube formation between HPMCs

**Figure 1:**
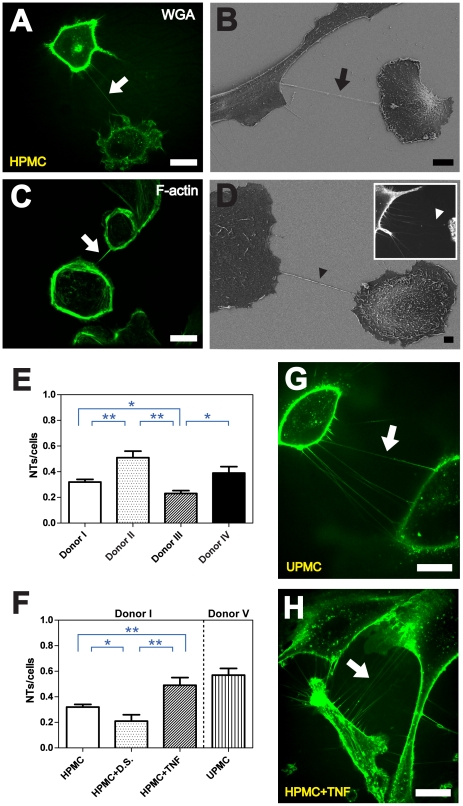
NT formation between HPMCs. (**A**) High resolution 3D live-cell fluorescence image of a NT (white arrow) connecting two primary mesothelial cells one hour after plating on a collagen I coated glass cover slide. To facilitate detection, cell membranes were stained with WGA Alexa Fluor® 488. Scale bar: 20 µm. (**B**) Depiction of a NT (black arrow) between two cells with scanning electron microscopy one hour after cell plating. Scale bar: 10 µm. (**C**) F-actin staining by fluorescently labeled phalloidin showing actin being present in NTs between individual HPMCs (white arrow). Scale bar: 20 µm. (**D**) Scanning electron microscope picture of a substrate-associated filopodia-like extension as potential NT precursor (black arrowhead). The insert shows a fluorescence microscopic image of substrate associated filopodia-like protrusions approaching a neighboring cell (white arrowhead). Scale bar: 2 µm. (**E**) Quantitative analyses of the NTs/cells ratio from 4 different donors undergoing abdominal surgery. (**F**) Influence of dialysis solution and TNF-α on NT formation between cells from Donor I. For comparison, the NTs/cells ratio is shown for UPMCs from a patient undergoing peritoneal dialysis (Donor V). (**G, H**) Live-cell fluorescence microscopy showing an increased number of NTs between cells from Donor I after TNF-α treatment (**G** arrow) and between UPMCs from Donor V one hour after cell plating (**H** arrow). Scale bars: 20 µm. All data are means ± SEM. * p<0.05, ** p<0.01 (*t*-test in **E**, **F**).

Since *in vivo* analysis of NTs in patients is unfeasible, we developed HPMC primary cultures from omentum obtained during abdominal surgery or from effluents of overnight bags from patients undergoing PD (Table S1). By applying fluorescence and scanning electron microscopy, we were able to detect thin membrane tethers, interconnecting individual HPMCs (Fig. 1). The structures were clearly distinguishable from filopodia or other classical cellular protrusions by being tensed between cells at their nearest distance and having no contact to the substratum (Fig. 1A and B). Immunolabeling of F-actin revealed the presence of actin fibers within the tubes (Fig. 1C). Both findings are in consistency with previous observations made for various other cell types [5], [11], [12]. Frequently, discrete filopodia-like protrusions in contact with the substratum and seemingly directed towards adjacent cells, emitted by individual HPMCs were observable (Fig. 1D).

To elucidate, whether the observed structures permit the exchange of cellular material, we performed microinjection experiments by injecting fluorescently labeled dextran in one cell of a NT connected cell-pair. 45 min after injection, dye signals were detectable in the non-injected cell (Fig. S1). This connectivity of HPMCs may point to complex communication processes in the peritoneum, involving supplement and/or rescue functions in order to maintain tissue integrity. Analogue supportive functions were discussed for NT development in rat hippocampal astrocytes and neurons, which – in contrast to HPMCs (data not shown) - seems to be largely dependent on p53 activation [13].

### Influence of TNF-α

By determining the number of NTs between HPMCs arising from 4 patients, each manifesting a different pathological background (Table S1), significant differences became obvious (Fig. 1E). Regarding the same tissue, this raised the question, whether varying NT numbers are based on individual variations, *e.g.* in relation to specific diagnostic findings. We therefore investigated uremic peritoneal mesothelial cells (UPMCs) derived from four patients (Donor V, Donors VII–IX) undergoing PD and found increased NT numbers (Fig. 1F and G, Fig. S2). To test, whether this increase relates to inflammatory reactions, we treated HPMCs from the non-uremic Donor I with soluble TNF-α as well as dialysis solution. This showed that TNF-α led to strongly increased NT numbers (Fig. 1F and H) comparable to the situation found for UPMCs. In contrast, incubation with dialysis solution resulted in a significant decrease of NT numbers (Fig. 1F).

**Figure 2:**
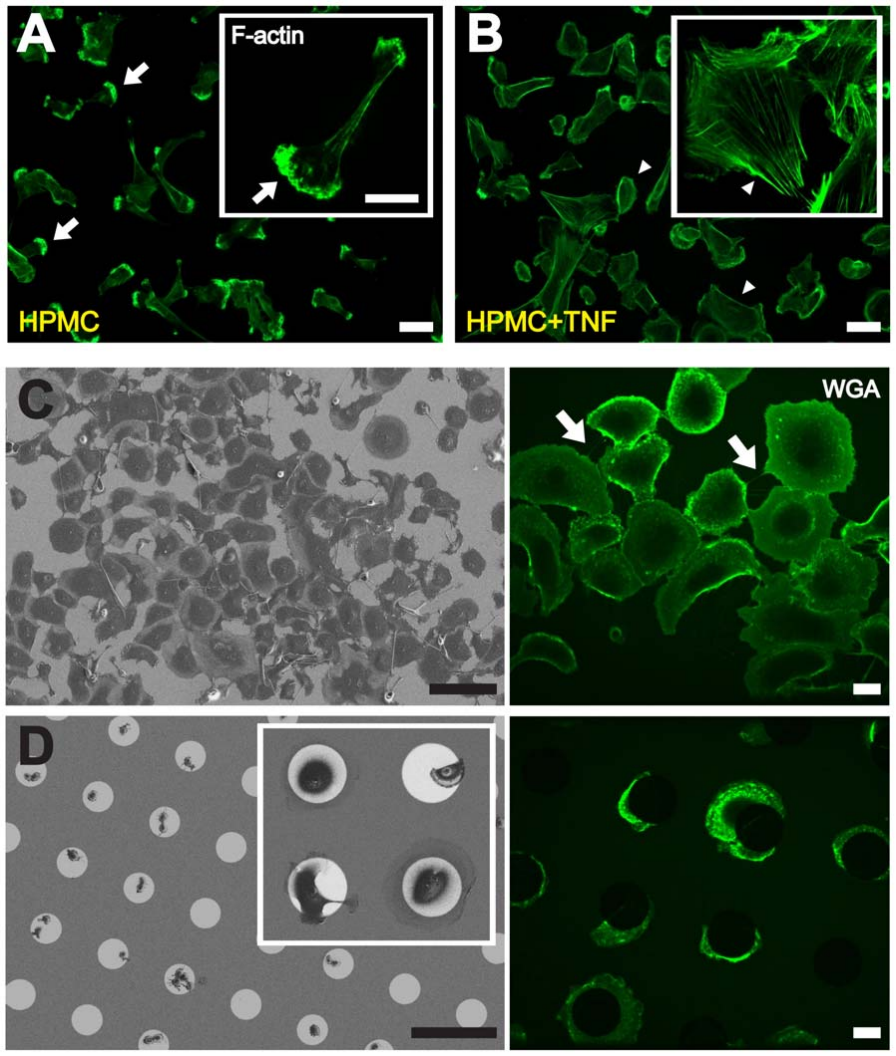
**(A, B)** Influence of TNF-α on actin cytoskeleton reorganization in HPMCs. (**A**) The fluorescence picture shows a predominant localization of F-actin at the rim of the cells (arrows) in absence of TNF-α. (**B**) The presence of TNF-α leads to reorganization of the actin cytoskeleton accompanied by the formation of actin stress fibers (arrowheads). Scale bars: 50 µm. (**C**, **D**) Spatial separation of HPMCs results in complete inhibition of NT formation. (**C**) Scanning electron and live-cell fluorescence microscopy pictures of HPMCs one hour after plating on homogeneously collagen I coated glass cover slides. The white arrows point to NTs spanned between the cells. Scale bars (left): 100 µm; (right): 20 µm. (**D**) HPMCs seeded on IKVAV functionalized (50 µM) microstructured surfaces with a gold disk diameter and spacing of 50 µm. Scanning electron microscopy (left panel) and live-cell imaging analyses (right panel) were performed one hour after plating. Note that cells exclusively attach to the biofunctionalized gold disk structures. The inserts in **A, B** and **D** represent higher magnified images. Scale bars (left): 100 µm; (right): 20 µm.

To approach the mechanism by which TNF-α affects nanotube formation, we analyzed its influence on the cytoskeleton. In this respect, it was shown that TNF-α action can lead to an accentuation and redistribution of actin microfilaments [14], increased stress fibers [15] and the rapid development of extending filopodia [16]. To test whether the cytokine evokes analogous effects in HPMCs, we cultured cells (Donor I) in the absence or presence of TNF-α (Fig. 2A and B). In its absence, actin was located mainly in the cell cortex with apparent culmination at the leading edges (Fig. 2A). In contrast, prominent stress fibers were detectable in the presence of TNF-α, accompanied by less elongated cell morphologies and a nearly complete loss of the leading edge signals (Fig. 2B). Along with the presence of F-actin inside the NTs, the stimulating effect of TNF-α may be caused – directly or indirectly - by a remodeling of the actin cytoskeleton, in turn, affecting filopodia activity.

To support this notion, we employed microstructured gold disk surfaces with precisely defined structural parameters. Similar surfaces were used to “trap” cells at given patterns, efficiently preventing them from migrating [12]. In comparison to the cultivation on standard cell culture dishes (Fig. 2C), HPMCs exclusively adhered to the functionalized disk structures (Fig. 2D). Remarkably, NT formation was almost completely abolished (Fig. 2D). This shows that spatial separation inhibits NT-formation between HPMCs, presumably by prohibiting the formation of filopodia by the adhesion repellent area between the gold disks.

### Impact of plasma membrane composition on NT formation

**Figure 3:**
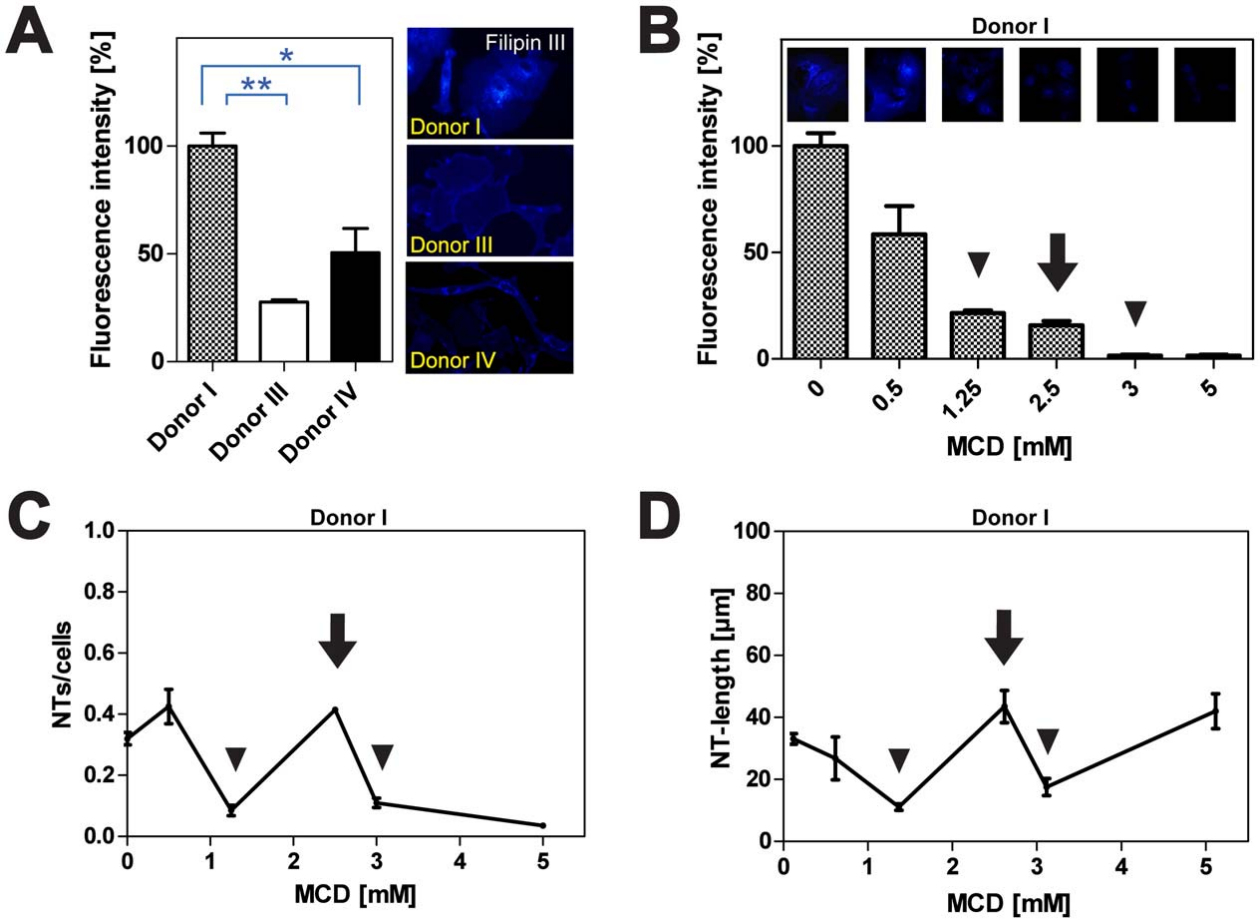
Impact of MCD mediated cholesterol depletion on NT formation. (**A**) Quantitative analyses of fluorescence intensities after cholesterol staining with Filipin III in HPMCs from different donors (left panel). Corresponding fluorescence images of stained cells one hour after cell plating are shown (right panel). (**B**) HPMCs were cultured in absence or presence of indicated MCD concentrations and Filipin III staining was performed one hour after plating. For quantification, fluorescence intensities based on representative microscopic images (top row) were determined as described. Please note that cholesterol depletion results in a decrease of fluorescence intensities (arrowheads, arrow). (**C**) Analysis of the NTs/cells ratio dependent on cholesterol depletion. HPMCs were treated with indicated MCD concentrations and NT numbers were assessed one hour after cell plating. The graph shows a significantly higher number of NTs when cells are treated with 2.5 mM MCD (arrow) as compared to the control or treatment with 1.25 or 3 mM MCD (arrowheads). (**D**) Quantitative analyses of NT lengths after cholesterol depletion. HPMCs were cultured in the presence of indicated MCD concentrations and NT lengths were analyzed one hour after cell seeding. Incubation of cells with 1.25 mM MCD led to comparatively short NT lengths (arrowhead) whereas incubation with 2.5 mM MCD led to extended NT lengths (arrow). All data are means ± SEM. * p<0.05, ** p<0.01 (*t*-test in **A**).

Apart from the actin/filopodia based formation process, little is known about NT regulating effectors. Since an influence of the enwrapping lipid coat cannot be excluded, we investigated the impact of plasma membrane composition. For this purpose, the cholesterol content of HPMCs from Donors I, III and IV was assessed by Filipin III staining (Fig. 3A). This revealed that the fluorescence intensity was significantly higher in cells from Donor I when compared to Donors III and IV (Fig. 3A). Besides varying cholesterol contents, striking differences in its cellular distribution became apparent (Fig. 3A). Whereas for Donor I the signal was located mainly intracellular, for Donor III a nearly exclusive localization at the plasma membrane was observable. For cells from Donor IV a homogenous distribution was found.

We next performed experiments to affect cellular cholesterol homeostasis. As a start, we employed cholesterol depletion *via* methyl-β-cyclodextrin (MCD) to quantitatively alter cholesterol concentrations in cells from Donor I (Fig. 3B). The results show that with increasing MCD concentrations a gradual decrease in Filipin signal intensity was observable, indicative of declining amounts of cellular cholesterol (Fig. 3B). To test, whether the varying cholesterol contents affect NT formation, comparable experiments addressing NT numbers were performed. These revealed that gradual cholesterol depletion results in a strong, non-linear modulation of NT numbers with significant peaks at 0.5 and 2.5 mM MCD (Fig. 3C). In parallel, we analyzed the length distribution of the NTs, which could provide a measure for their stability. As evident for NT numbers, modulation of the cholesterol content resulted in non-linear lengths variations with a prominent peak at 2.5 mM MCD (Fig. 3D).

**Figure 4:**
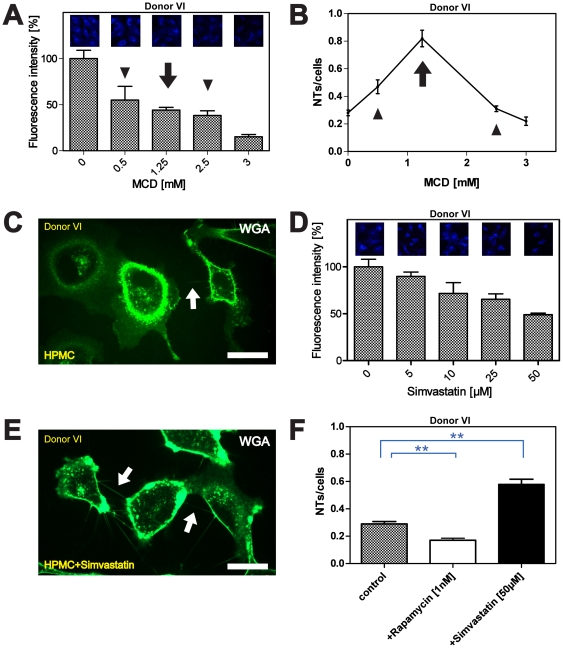
Influence of MCD and simvastatin on NT formation between HPMCs. (**A**) HPMCs from Donor VI were cultured in absence or presence of indicated MCD concentrations and Filipin III staining was performed one hour after plating. Cholesterol depletion results in a decrease of fluorescence intensities (arrowheads, arrow). (**B**) Analysis of the NTs/cells ratio dependent on cholesterol depletion. HPMCs were treated with indicated MCD concentrations and NT numbers were assessed one hour after cell plating. The graph shows a significantly higher number of NTs when cells are treated with 1.25 mM MCD (arrow) as compared to the control or treatment with 0.5 mM and 2.5 mM MCD (arrowheads). (**C**) Live-cell fluorescence microscopy showing NTs (arrow) between the cells under control conditions one hour after cell plating. Scale bar: 20 µm. (**D**) Quantitative analyses of fluorescence intensities after cholesterol staining with Filipin III. Filipin staining was performed in the presence of indicated concentrations of simvastatin as described. For quantification, fluorescence intensities based on representative microscopic images (top row) were determined as described. Increasing concentrations of simvastation resulted in a linear decrease of fluorescence intensities. (**E**) Live-cell fluorescence microscopy after simvastatin treatment revealed strongly elevated NT numbers between cells (arrows) as compared to the control condition. (**F**) Quantitative analyses of the NTs/cells ratio from Donor VI after treatment with rapamycin or simvastatin. Cells were cultured in absence (control) or presence of indicated rapamycin or simvastatin concentrations and NT numbers were analyzed one hour after cell seeding. Incubation of cells with 1 nM rapamycin led to a significant decrease of NT numbers (white bar) whereas incubation with 50 µM simvastatin resulted in a strong increase (black bar). All data are means ± SEM. ** p<0.01 (*t*-test in **F**).

To exclude that the non-linear nature of the observed results is based on unique features of the cells derived from Donor I, we performed equivalent experiments with HPMCs from Donor VI. The results showed that again a non-linear correlation of NT numbers with decreasing cholesterol contents was detectable (Fig. 4A and B). This time one peak at 1,25 mM MCD was observable (Fig. 4B). The shifted peak position may point to subtle individual variances, potentially connected or in analogy to the differences observed for cholesterol localization (compare Fig. 3A).

As a second approach to influence cholesterol homeostasis, we employed the therapeutical drug simvastatin, an inhibitor of HMG-CoA reductase (Fig. 4D–F). The experiments show that the treatment of cells from Donor VI with varying simvastatin concentrations led to a gradual decrease of Filipin intensities (Fig. 4D). Interestingly, simvastatin treatment was accompanied by a strong induction of NT numbers (Fig. 4E and F) as compared to the control situation (Fig. 4C). The use of the immunosuppressant drug rapamycin under the same conditions led to a significant decrease of NT numbers between the cells (Fig. 4F).

## Discussion

Taken together, we show that primary HPMCs establish discrete nanotubular connections and provide support for an actin/filopodia dependent *de novo* formation process as demonstrated for other cell systems [17]. The finding that TNF-α boosted NT formation between HPMCs shows striking parallels to studies with other immune-relevant cells or *in* vivo systems [8], [10], *e.g.* showing that dendritic cells in the mouse cornea formed more nanotubes on stimulation with lipopolysaccharides or mechanical injury [10]. Together, these findings point to a basal participation of NTs during inflammatory reactions. This notion gains further importance in view of studies demonstrating the misuse of NTs by different pathogens, such as prions [18] or viruses [7], [19].

Furthermore our results clearly show that there is a strong correlation of NT occurrence with HPMC cholesterol contents. In particular the steep increase/decrease at given MCD concentrations, paralleled by increasing/decreasing NT lengths, points to narrow windows of defined cholesterol contents, which are beneficial or detrimental, *e.g.* by affecting NT tensile strength *via* altered membrane fluidity or by influencing the formation process in itself. With respect to the individual physiopathological differences found for cholesterol localization (Fig. 3A) and the individually defined cholesterol contents leading to culminating TNT numbers (compare Fig 3C and 4B) the observed effects are likely to be correlated not exclusively to the mere cholesterol content, but also to its distribution throughout the cell and thus, to the accountable transport machinery. In this view, it is interesting to note that lipid metabolic pathways are known to be involved in several diseases [20].

In conclusion, our results demonstrate a complex interdependence of NT mediated cell communication, cytokine action and cholesterol homeostasis, which will have significant impact for the understanding of a variety of processes, such as inflammatory immune reactions. Given that our findings for primary mesothelial cells are transferable to the *in vivo* situation, they may urge to reconsider *e.g.* therapeutic sanctions, such as the application of statins, which may have to be precisely adopted to the individual patient background, in order to 1) induce potential beneficial effects and 2) not to provoke undesired reverse effects. In support of this notion it was shown, that statin treatment can be associated with improved survival [21]–[24] and anti-inflammatory effects in peritoneal dialysis patients [25], [26]. Such potential interrelations will now have to be approached in great detail, whereby several problems, *e.g.* access to relevant tissues/cells derived from patients have to be faced.

## Materials and Methods

### Cell preparation, cultivation and characterization

Peritoneal tissue was obtained from five patients during abdominal surgery. Human peritoneal mesothelial cells (HPMCs) were isolated from portions of omentum or from overnight bags of four patients undergoing peritoneal dialysis. The study was approved by the institutional ethic committee of the Medical Faculty, University of Heidelberg, and a written informed consent was given by all participating patients (Vote numbers 089/2004 and S-461/2010). For the isolation of HPMCs from omentum, pieces were washed twice in sterile phosphate buffered saline (PBS) and cut into portions of approximately 2 cm^2^. To disaggregate the tissue, the pieces were incubated in a trypsin/EDTA-solution (0.025%/0.01% (w/v), PAA) in PBS in a 50 ml centrifuge tube under continuous stirring for 30 min at 37°C. After the incubation, cells were centrifuged (1200 rpm, 4°C, 10 min) and seeded in 25 cm^2^ cell culture flasks coated with collagen type I (Greiner Bio-One) containing M199 medium supplemented with 10% fetal calf serum (FCS), 2 mM L-glutamine (all PAA), insulin/transferrin [4 µg/ml each] (Gibco) and hydrocortisone [0.4 µg/ml] (Sigma-Aldrich). For the isolation of HPMCs from overnight bags, the effluent is collected and spinned in 50 ml centrifuge vials for 10 min at 4°C and 1800 rpm. After removal of the supernatant, the pellet is suspended in PBS and pooled to 15 ml vials and centrifuged as described. The supernatant was removed, the pellet is suspended in cell culture medium and the cells are seeded in 25 cm^2^ cell culture flasks coated with collagen type I (Greiner Bio-One). The cells were cultured at 37°C in 5% CO_2_. After reaching confluence, cells were trypsinized (trypsin/EDTA 0.05%/0.02% (w/v), PAA) and diluted in fresh medium. For characterization, cells were immunocytochemically stained with antibodies for cytokeratin 18 (Millipore) and human fibroblast surface protein (Sigma-Aldrich). All experiments presented were performed with cells from the first to the third passage. For microscopy experiments, 6×10^4^ cells were plated on the respective surfaces glued underneath a 35 mm cell culture dish in which a hole with a diameter of 14 mm had been cut.

### Generation and biofunctionalization of micropatterned surfaces

Microstructured and biofunctionalized gold disc surfaces were prepared by means of photolithography as previously described [12]. In brief, glass coverslips (20×20 mm, Carl Roth GmbH) were coated with a positive photoresist AR-P 5350 (Allresist GmbH) and overlaid with a chromium mask produced with a maskwriter (DWL-66, Heidelberg Instruments Mikrotechnik GmbH), performed on a mask aligner (MJB-3, Karl Suess MicroTec Lithography GmbH). The photoresist was solubilised after exposure to a mercury lamp in an alkaline developer AR300-35 (Allresist GmbH). A 7 nm titanium and a 25 nm gold layer were sputtered on the coverslips by a sputter coater (MED020, Bal-Tec GmbH) such that the metal layers were thin enough to visualize the plated cells on the gold discs by light microscopy. The “lift-off” procedure was performed by the use of acetone *p.a.* and dimethylformamide *p.a.* (Merck).

For biofunctionalization, microstructured surfaces were cleaned 5 min in hydrogen plasma (E-100, PVA TePla). To couple IKVAV peptide (Bachem) covalently on the gold discs, surfaces were incubated for at least 12 hours at 4°C in a 50 µM IKVAV solution in PB buffer. Samples were rinsed three times with PB buffer for 5 min on a shaker and were used immediately or stored until use in PB buffer at 4°C.

### High resolution scanning electron microscopy (SEM)

For SEM, cells were fixed with 2.5% glutaraldehyde and 0.1 M sodium cacodylate (pH 7.4), dehydrated in a graded series of ethanol and critically point dried by the use of CO_2_ (Critical Point Dryer CPB030, BalTec). To visualize the cells on the surfaces, the samples were coated with a 5 nm thick layer of graphite (7×10^−5^ mbar pressure, BAL-TEC MED020 Sputter System, BalTec) and analyzed with an in-lense field emission Scanning Electron Microscope (ZEISS LEO 1530, Carl Zeiss NTS GmbH). The generated images were analyzed with Image J software (version 1.44o) (European Molecular Biology Laboratory).

### Conventional fluorescence microscopy

Fluorescence microscopy was performed with a Nikon Eclipse 80i microscope equipped with a Nikon Plan Fluor 20x NA 0.50 objective (Nikon GmbH). Image acquisition and processing were controlled by NIS Elements BR 3.0 software. The images were visualized and processed with Image J software (version 1.44o) (European Molecular Biology Laboratory).

### Spinning disc microscopy

Confocal microscopy was performed on a Perkin Elmer Ultraview system (Perkin Elmer Inc.) mounted on a Nikon TE 2000E inverted microscope with Nanoscan z-device, equipped with Nikon Plan Apo VC 100x NA 1.4 or VC 60x NA 1.4 oil-immersion planapochromatic objectives (Nikon GmbH) and surrounded by a 37°C heating chamber with CO_2_ regulation (Tokai Hit Microscope Incubation System). The system uses a Perkin Elmer spinning disc confocal ERS-FRET Unit (laser lines: 405, 488, 568 nm) in combination with CFP-YFP, GFP-mRFP and CFP-mRFP filter sets (Perkin Elmer Inc.). Pictures were taken with a 30 Hz charge-coupled device camera (EM-CCD, Hamamatsu Photonics Deutschland GmbH). For 3D analysis, a piezo z-stepper (Perkin Elmer Inc.) was used. Camera and z-stepper were controlled by the UltraVIEW ERS software system. Images were processed with the Perkin Elmer Volocity acquisition software (Version 5.4.1).

### Microinjection

For microinjection experiments, 6×10^4^ cells were plated on collagen type I coated glass cover slips glued underneath a 35 mm cell culture dish as described. After one hour of incubation, selected cells were injected with 1 µl Dextran Texas Red® (MW 10000; Molecular Probes), which had been loaded into a microinjection needle (Femtotips II, Eppendorf AG), using a micromanipulation system (FemtoJet®; Eppendorf AG) mounted on a fluorescence microscope equipped with a 37°C heating chamber (Leica DM IRB, Leica Microsystems).

### Reagents and dye staining

Human soluble TNF-α was kindly provided by Prof. Dr. P. Scheurich (Institute of Cell Biology and Immunology, University of Stuttgart) and used for the experiments at a concentration of 100 ng/ml. Gambrosol trio 10 supplemented with 2.5% glucose was purchased at Gambro Lundia AB, Lund, Sweden. For cell culture experiments, a 1∶1 ratio of cell culture medium and dialysis solution was used. Rapamycin (Sigma-Aldrich) was dissolved in 100% Ethanol to a 2 mM solution and used for cell culture experiments at a concentration of 1 nM. For plasma membrane staining, WGA Alexa Fluor® 488 conjugate (Invitrogen) at a concentration of 1 mg/ml was added directly to the cell culture medium. For cholesterol depletion experiments, a 330 mM Methyl-β-cyclodextrin (Sigma-Aldrich) solution in DMSO was prepared. The solution was added to the cell culture medium such that final MCD concentrations of 0.5 mM, 1.25 mM, 2.5 mM, 3 mM and 5 mM were achieved. After incubation for one hour, the cell culture medium was removed and cells were fixed with 4% PFA in PBS for 5 min at room temperature. After extensive washing, cells were incubated with a 10 µM Filipin III (*Streptomyces filipinensis*, Sigma-Aldrich) solution in PBS for 30 min at room temperature. After washing, samples were mounted with *SlowFade*® Gold antifade reagent (Invitrogen) for image processing. Simvastatin (Sigma-Aldrich) was activated before use by dissolving 4 mg in 100 µl of 100% ethanol and 150 µl of 0.1 N NaOH. The solution was heated at 50°C for 2 h, then the pH was adjusted to 7.2 with HCl and the final volume was set to 1 ml. The stock solution was diluted to concentrations of 5 µM, 10 µM, 25 µM and 50 µM in cell culture medium containing 1% FCS immediately before use. After incubation for one hour, live-cell-imaging and Filipin III staining were performed as described. Cytoskeleton was labeled using a 500 x diluted solution of Alexa Fluor 488 phalloidin (Invitrogen). Cells were fixed as described above, incubated one hour at room temperature, washed and mounted for image processing. For p53 staining, cells were fixed as described and incubated for 1 h at room temperature with a monoclonal mouse antibody specific for p53 of human origin (IgG_2a_; 200 µg/ml, Santa Cruz; dilution 1∶100 in PBS). After incubation, samples were rinsed three times with PBS and treated with an Alexa 488-coupled mouse IgG-specific secondary antibody (Sigma-Aldrich; dilution 1∶1000) for 1 h at room temperature. Again, samples were rinsed as described above and mounted before fluorescence microscopy.

### Statistical analyses

For quantification of NT-numbers, all statistical data represent the mean of three independent experiments. In each experiment, twenty randomly selected fields each containing approximately 5 cells were analyzed. Fluorescence intensity quantification in cholesterol staining experiments was performed using Image J software (version 1.44o). For the analyses, three randomly selected fields of one surface were used. Graphs were generated with GraphPad Prism 5 software (Statcon). Error bars represent the standard error of the mean. The calculation of statistical significance was performed by the use of student's t-test (Origin 6.1 software, OriginLab Corporation) in which the level of significance was set to p<0.05 and p<0.01.

## Supporting Information

Figure S1 **Assessment of an exchange of cellular material between NT-connected HPMCs by microinjection.** (**A**) Cell membranes were stained with WGA 488 (green) and for injection of fluorescently labeled dextran Texas Red® (red) one cell of a NT-connected cell-pair was selected (asterisk). The arrow points to the connecting NT. (**B**) Merged fluorescence picture showing the injected cell 5 min after injection. (**C**) Flow of the dye into the NT 10 min after injection (arrowhead). (**D**) 45 min after injection, the dye was detectable in the non-injected cell (arrowheads). Scale bars: 20 µm.
(TIF)

Figure S2 **NT numbers between HPMCs from CAPD-patients.** Quantitative analyses of the NTs/cells ratio from 3 different individuals (Donors VII–IX) undergoing CAPD.
(TIF)

Table S1 Patient characteristics. Peritoneal biopsies from 5 individuals (Donors I–IV, VI) and overnight bags from four PD-patients (Donor V, VII–IX) were collected as described. Details concerning age, gender, reason for surgery, tumors, diabetes and infections/sepsis of the patients are listed.
(TIF)
