# Supplementary figures and images for: Nanotube Action between Human Mesothelial Cells Reveals Novel Aspects of Inflammatory Responses
Source: PLoS One. 2011 Dec 27;6(12):e29537. doi: 10.1371/journal.pone.0029537 (PMC3246504; doi:10.1371/journal.pone.0029537)

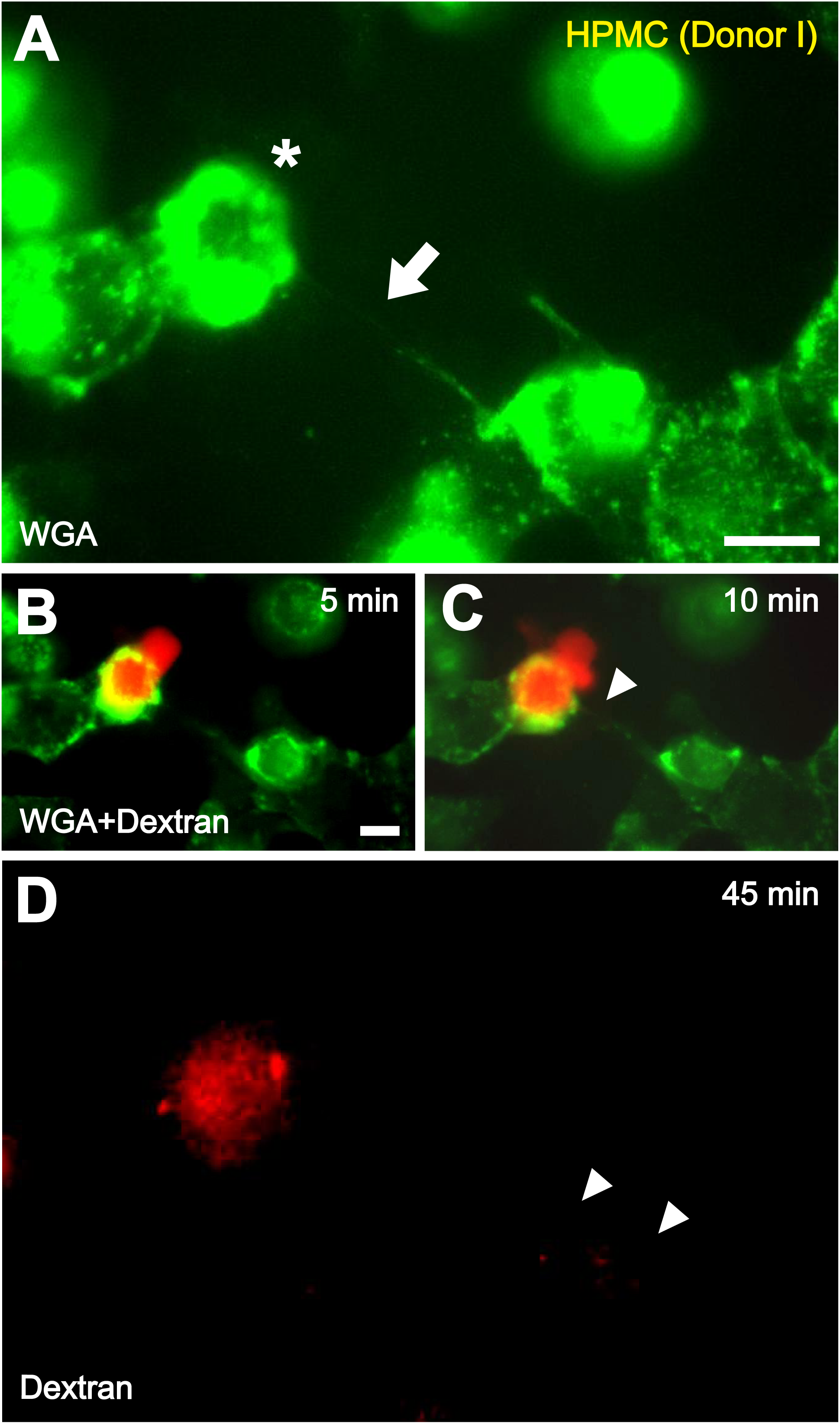

Supplement: Figure S1 — Assessment of an exchange of cellular material between NT-connected HPMCs by microinjection. (A) Cell membranes were stained with WGA 488 (green) and for injection of fluorescently labeled dextran Texas Red® (red) one cell of a NT-connected cell-pair was selected (asterisk). The arrow points to the connecting NT. (B) Merged fluorescence picture showing the injected cell 5 min after injection. (C) Flow of the dye into the NT 10 min after injection (arrowhead). (D) 45 min after injection, the dye was detectable in the non-injected cell (arrowheads). Scale bars: 20 µm. (TIF) [file pone.0029537.s001.tif]

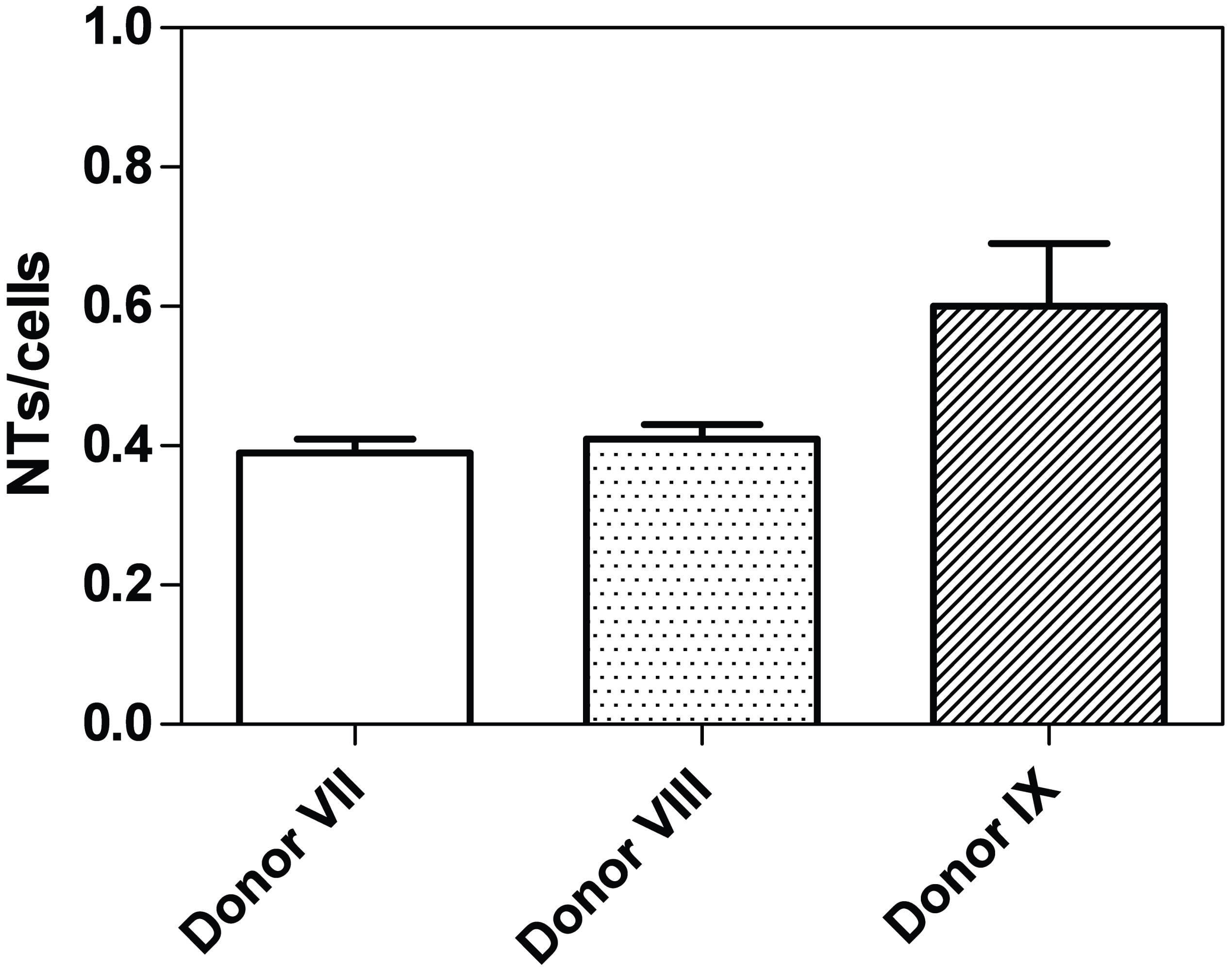

Supplement: Figure S2 — NT numbers between HPMCs from CAPD-patients. Quantitative analyses of the NTs/cells ratio from 3 different individuals (Donors VII–IX) undergoing CAPD. (TIF) [file pone.0029537.s002.tif]

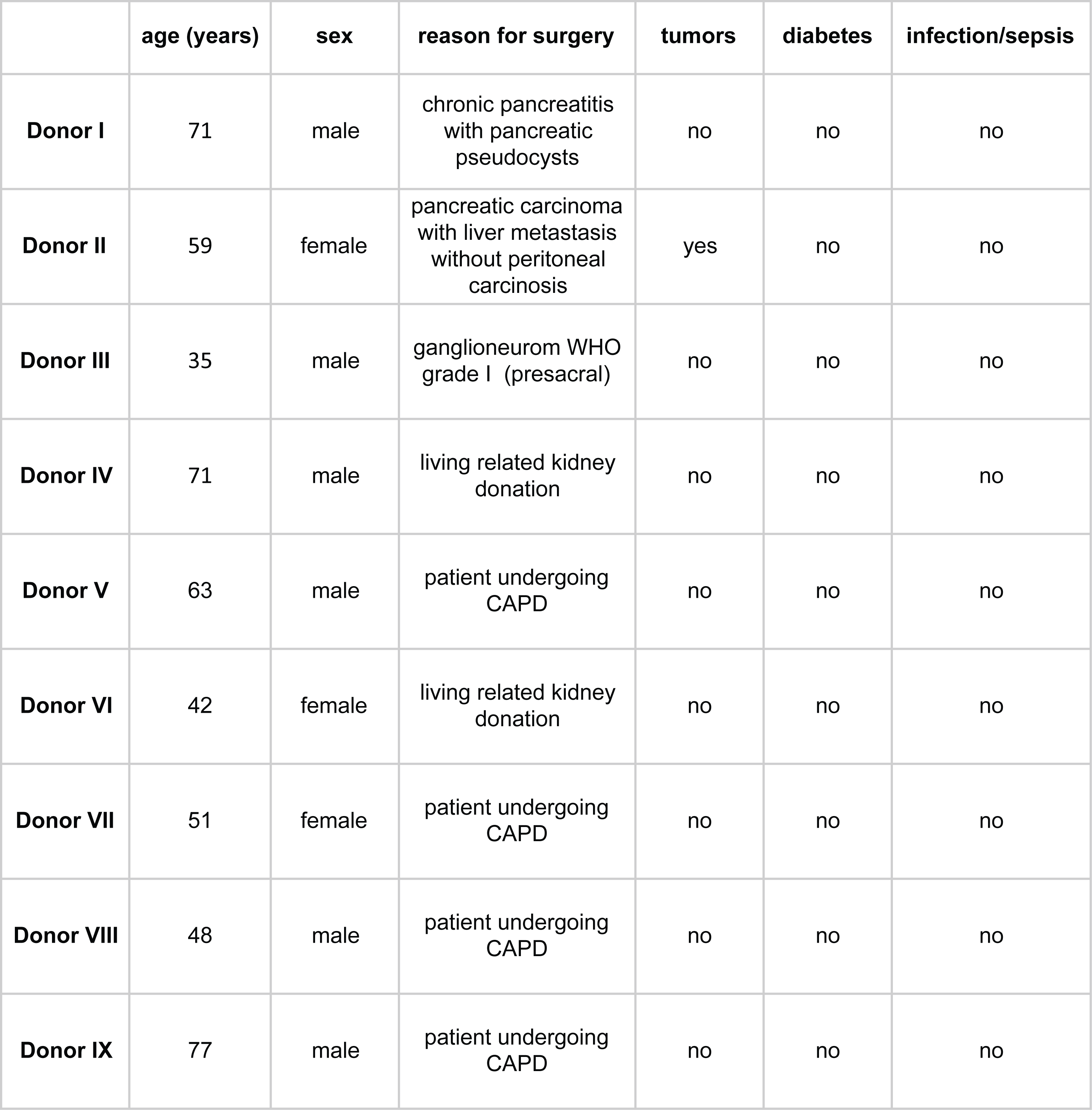

Supplement: Table S1 — Patient characteristics. Peritoneal biopsies from 5 individuals (Donors I–IV, VI) and overnight bags from four PD-patients (Donor V, VII–IX) were collected as described. Details concerning age, gender, reason for surgery, tumors, diabetes and infections/sepsis of the patients are listed. (TIF) [file pone.0029537.s003.tif]
